# Prevalence and correlates of diarrhoea among children under five in selected coastal communities in Ghana

**DOI:** 10.1186/s41043-024-00582-8

**Published:** 2024-06-26

**Authors:** Delia Akosua Bandoh, Duah Dwomoh, Dzidzo Yirenya-Tawiah, Ernest Kenu, Mawuli Dzodzomenyo

**Affiliations:** 1 School of Public Health, University of Ghana, Legon, Accra, Ghana; 2 Institute for Environment and Sanitation Studies, University of Ghana, Legon, Ghana

**Keywords:** Diarrhoea, Coastal community, WASH, Health seeking behaviour

## Abstract

**Introduction:**

Diarrhoea is a preventable disease affecting children under five years disproportionately. Globally, thousands of children die from diarrhoea related diseases each year, most deaths occuring in sub-Saharan Africa where Ghana is located. Coastal communities bear the greatest brunt due to poor sanitary conditions. We assess the prevalence of diarrhoea in selected coastal communities along the eastern coast of Ghana.

**Methods:**

We conducted a cross-sectional study in Mumford, Opetekwei, Anyako, Anyauni and Ateteti communities in the Central, Greater Accra and Volta region respectively. We interviewed households with children under five years on the occurrence of diarrhoea and health seeking practices. We also used a checklist to assess the sanitary conditions of the household. Frequencies and proportions were generated. We determined significant differences using modified Poisson regression models at *p* < 0.05. Results were presented in tables and text.

**Results:**

The prevalence ratio of diarrhoea was 36% (95% CI 33–40%). Most cases were from Anyako community. All interviewed households in Mumford and Opetekwei used improved water sources whiles 94% in Atetetio used improved water sources. Children who were fully vaccinated had 32% lower prevalence of diarrhoea compared to those who were not (aPR: 0.68, 95% CI 0.55–0.84).

**Conclusion:**

Diarrhoea prevalence was high inspite of the reported use of improved water sources and sanitation facilities by majority of households in the communities. Fully vaccinated children had a relatively lower prevalence of diarrhoea compared to children who were not fully vaccinated. We recommend in-depth analysis of the use of water and sanitation facilities in these settings to understand the reasons for the observed diarrhoea prevalence.

## Introduction

Diarrhea is a climate sensitive disease disproportionately affects children under five years and remains one of their second leading cause of deaths globally [1, 2]. In 2019, almost 1.7 billion children under five years developed diarrhoea with 525,000 deaths [3]. The greatest burden of the diarrhoea menace was borne by low and middle income countries (LMICs) where improved water, sanitation and hygiene remain a consistent problem. In Ghana, diarrhoea forms part of the ten top causes of children under five years in-patient morbidity [4] with an estimated prevalence of about 13% among children under five years as at 2022 [5].

Unfortunately, the relative risk of diarrhoea is projected to increase by about 8–11% by the next decade due the adverse impact of climate change [6]. This puts countries like Ghana located in sub- Saharan Africa (SSA) at risk since they bear the greatest burden of climate attributable deaths [7]. To confirm this, a survey by Abu and Cudjoe [8] found a perception of high risk of flooding and the incidence of diarrhoeal disease in a coastal urban area in Ghana.

Ghana has a coastal line cutting across the entire southern part of the country. Similar to other coastal communities, the Ghanaian coast is characterised by dense populations with poor infrastructure [9]. Generally, coastal communities are more prone to climatic change consequences due to their proximity to the sea and sensitivity to sea level rise and remain more vulnerable to the detrimental effects of climate change [10]. Again, coastal areas are densely populated and have a poorly planned layout making them prone to poor sanitary conditions and vulnerable to poor WASH practices [11]. A study conducted in selected coastal communities in Accra found the communities to be vulnerable to flooding during increased rainfall and water scarcity during decreased rainfall [11] both situations are precursors of poor sanitation and increase the incidence of diarrhoea in these communities.

Several other studies have associated diarrhoea in children under five to poor water sanitation and hygiene factors which lead to ingestion of contaminated food and water [12–15]; Socio-demographic factors including wealth index, caregiver educational level, and environmental factors which influence child feeding and caregiving practices [16–19]. Diarrhoeal disease reported as outpatient morbidity in Ghanaian health facilities, increased from 287,816 cases in 2012 to 1,429,990 in 2017 [4]. Like other developing countries, diarrhoeal disease transmission in Ghanaian settings have been associated with unsafe water, poor sanitation and hygiene practices [20].

With climate change already impacting negatively on the coastal areas of Ghana in the form of erosion, floods and storms, the transmission environments of diarrhoea diseases are expected to be influenced. Other factors that expose populations at risk of diarrhoea are poor sanitation, poverty, contaminated food and water. Therefore, understanding the diarrhoea pathways in coastal communities in Ghana is important in the reduction of diarrhoea diseases in these areas and the country as a whole. Therefore, understanding the diarrhoea pathways in coastal communities in Ghana is important in the reduction of diarrhoea diseases and implementation of climate resilient interventions which can reduce the impact of climate change on these communities. We assessed the prevalence of diarrhoea in selected coastal communities along the eastern coast of Ghana and associated factors.

## Methods

### Study design

We conducted an analytic cross-sectional study in selected coastal communities in Ghana to estimate the prevalence of diarrhoea among children under-fives years and factors influencing diarrhoea cases these communities. This was done through household interviews with caregivers of children under five years. Data was collected from July to September 2021.

### Study site

The study was conducted in coastal communities along the eastern and central coasts of Ghana. namely; Opetekwei in the Greater Accra region, Mumford in the Central region, Anyako, Anyanui and Ateteti in the Volta regions of Ghana. Communities were peri-urban in nature with Opetekwei being an urban slum. Communities were purposively selected due to their proximity to the water bodies and their risk of climate change consequences such as flooding.

### Study population

All caregivers living in the study communities with a child under five years were eligible to partake in the study population for the survey. We included all caregivers of children under 5 years (0–59 months) living in Anyanui, Anako, Ateteti, Mumford and Opetekwei during the survey period. We excluded caregivers who were unavailable in their houses at the time of interview.

### Study variables

Outcome variable: Occurrence of diarrhoea in a child under five years over the past two weeks. We defined diarrhoea using the WHO standard definition, the passage or two to three watery stools in a day [21]. The caregiver was asked whether their child had had diarrhoea over the past two weeks. The reference period was adopted from the demographic and health survey [16].

Independent variable: The independent variables were WASH practices, socio-demographic characteristics (age of care giver, educational level, occupation), health seeking practices (source of health education, point of call when child is unwell, vaccination status of child).

### Sample size determination

The sample size was calculated with the Cochrane formula using the prevalence of diarrhoea (11) from the Ghana Demographic and Health survey [20], a relative precision (d) = 0.05, a confidence interval at 95%$(Z_{{1 - \alpha /2}} )$) = 1.96 and a design effect ($Deff$) = 1.1.

$${\text{Minimum Sample size }}\left( {\text{n}} \right) = \frac{{ (Z_{1 - \alpha /2} )^{2} p\left( {1 - p} \right)}}{{d^{2} }}*Deff$$

An estimated sample size of 662 was obtained and rounded off to 680. The sample size allocated for each community was done through proportionate to size sampling; Opetekwei—170, Mumford—320, Anyako—102, Anyanui—44 and Atiteti—44.

### Sampling procedure/approach

Households were selected using the modified random walk method, a method used in a study among a similar population in coastal Ghana [22]. In the modified random walk, key land marks in the community were identified and one randomly selected as the starting point for the interviews. The first house on the right of the landmark was chosen as the first household to be interviewed. Subsequently, households were selected in a clockwise manner till the sample size for the community was reached. In each housing structure, only one household was interviewed. In a household with more than one child under five years, caregivers who were interested in taking part in the study balloted. The caregiver who picked ‘yes’ was interview. The modified random walk was used in all the five study communities till the sample size allotted to each community was achieved.

### Data collection techniques/tools

A structured questionnaire was administered to caregivers in their households. Information on socio-demographics of the household, health seeking behaviours, and WASH practices were obtained. Caregivers were also asked if their child had had a diarrhoea episode in the past two weeks. Questionnaires used for the interviews were adopted and modified from the DHS and MICS survey tools. Tools were pre-tested in a coastal community with similar characteristics as the study sites. The questionnaire was modified appropriately based on feedback from the pre-test.

### Data/statistical analysis

Data would be exported and cleaned in Microsoft Excel 2016. Imputation was done for missing data and cleaned data exported to STATA software version 14 for analysis. We classified wealth index, water and sanitation sources using the DHS statistics guide [23].

Water sources were classified as improved and unimproved. Piped water, Tube well / borehole, Protected well/ spring, Rainwater, Tanker-truck, Bottled water, Sachet water were classified as improved whiles Unprotected well, Unprotected spring, Surface water (river, dam, lake, pond, stream, canal, irrigation channel) were classified about unimproved. For sanitation facilities, all flush toilets, ventilated improved pit latrine, pit latrine with slab, Composting toilet, biodigester toilet were classified as improved sources whiles pit latrine without slab, open pit, Bucket, Hanging toilet / hanging latrine, No facility / bush / field and Beach/lagoon were classified as unimproved.

Wealth index was calculated using principal component analysis using water source, sanitation facility and ownership of the following household assets; radio, television, refrigerator, cabinet/cupboard, wristwatch, bank account, cooking fuel, and floor material. Wealth index was classified in five categories from poorest being the lowest to richest being the highest. Vaccination status was defined according to the age of the child, a fully vaccinated child was a child who had received all the age-appropriate vaccines according to the national immunization schedule for Ghana which include rotavirus vaccination given at 6 and 10 weeks respectively.

Summary statistics were be reported as frequencies and percentages for categorical variables. Prevalence of diarrhoea across the exposure variables was presented as percentages with corresponding 95% confidence interval. Modified Poisson regression model with robust standard error was used to estimate the effect independent variables on diarrhoea occurance. The effect size from the Modified Poisson regression model was reported in terms of Prevalence ratios with their correspondening 95% confidence interval. Level of significance was set at *p* < 0.05.

### Ethical consideration

The study was approved by the Ghana Health Service Ethic review committee (GHS-ERC 011/11/20). Permission was obtained from the respective community opinion leaders. An informed consent was obtained before nay interview was conducted. Participants were assured of their confidentiality throughout the interview and their voluntary participation in the study. Data obtained was without personal identifiers. All data obtained was stored on password protected servers accessible to selected members of the research team on basis of data analysis.

## Results

### Demographics

A total of 693 caregivers were interviewed across the five communities with a mean age of (35.15 ± 11.02) years. The average household size was 5 (± 2). Almost half (47%: 326/693) were traders (Tables 1, 2).

**Table 1:** Background characteristics of caregivers interviewed in coastal communities, 2021

Factor	Anyanui N = 47	Ateteti N = 46	Anyako N = 106	Opetekwei N = 170	Mumford N = 324	Total N = 693
Caregiver age						
18–29 years	17 (36.2)	12 (26.1)	39 (36.8)	36 (21.2)	113 (34.9)	217 (31.3)
30–39 years	20 (42.6)	20 (43.5)	43 (40.6)	91 (53.5)	125 (38.6)	299 (43.1)
40–49 years	4 (8.5)	8 (17.4)	13 (12.3)	26 (15.3)	55 (17.0)	106 (15.3)
50+	6 (12.8)	6 (13.0)	11 (10.4)	17 (10.0)	31 (9.6)	71 (10.3)
Number in household*						
Less than 5	24 (51.1)	21 (45.7)	38 (35.8)	79 (46.5)	127 (39.2)	289 (41.7)
5–10	22 (46.8)	24 (52.2)	67 (63.2)	90 (52.9)	180 (55.6)	383 (55.3)
More than 10	1 (2.1)	1 (2.2)	1 (0.9)	1 (0.6)	17 (5.2)	21 (3.0)
Wealth index**						
Poorest	16 (34.0)	28 (60.9)	34 (32.1)	2 (1.2)	71 (21.9)	151 (21.8)
Poor	6 (12.8)	5 (10.9)	24 (22.6)	16 (9.4)	78 (24.1)	129 (18.6)
Middle	6 (12.8)	7 (15.2)	21 (19.8)	17 (10.0)	85 (26.2)	136 (19.6)
Rich	9 (19.1)	4 (8.7)	20 (18.9)	38 (22.4)	68 (21.0)	139 (20.1)
Richest	10 (21.3)	2 (4.3)	7 (6.6)	97 (57.1)	22 (6.8)	138 (19.9)
Level of education of caregiver**						
No formal education	4 (8.5)	11 (23.9)	7 (6.6)	14 (8.2)	115 (35.5)	151 (21.8)
Primary	9 (19.1)	12 (26.1)	43 (40.6)	10 (5.9)	67 (20.7)	141 (20.3)
Middle/JHS	24 (51.1)	21 (45.7)	45 (42.5)	75 (44.1)	115 (35.5)	280 (40.4)
SHS/commercial	7 (14.9)	2 (4.3)	8 (7.5)	46 (27.1)	22 (6.8)	85 (12.3)
Tertiary	3 (6.4)	0 (0.0)	3 (2.8)	25 (14.7)	5 (1.5)	36 (5.2)
Primary occupation of caregiver**						
Unemployed	14 (29.8)	9 (19.6)	30 (28.3)	31 (18.2)	39 (12.0)	123 (17.8)
Formally employed	0 (0.0)	0 (0.0)	2 (1.9)	23 (13.5)	3 (0.9)	28 (4.0)
Fisherfolks	6 (12.8)	14 (30.4)	9 (8.5)	2 (1.2)	74 (22.8)	105 (15.2)
Trader	17 (36.2)	17 (37.0)	54 (50.9)	80 (47.1)	158 (48.8)	326 (47.0)
Artisan	10 (21.3)	6 (13.0)	11 (10.4)	34 (20.0)	50 (15.4)	111 (16.0)

*p < 0.05, **p < 0.001

**Table 2:** Prevalence of diarrhoea, by sociodemographic characteristics of respondents, 2021

Variable	Diarrhoea (%)	
	Yes (95% CI)	No
Caregiver age		
18–29 years	42.40 (35.98–49.08)	57.60
30–39 years	33.78 (28.63–39.34)	66.22
40–49 years	30.19 (22.21–39.58)	69.81
50+	35.71 (25.40–47.55)	64.29
Number in household		
Less than 5	33.56 (28.35–39.22)	66.44
05–10	37.70 (32.97–42.67)	62.3
More than 10	42.86 (23.99–64.06)	57.14
Wealth index		
Poorest	38.41 (30.99–46.41)	61.59
Poor	36.43 (28.58–45.08)	36.43
Middle	36.76 (29.08–45.19)	63.24
Rich	40.29 (32.45–48.65)	59.51
Richest	28.47 (21.53–36.59)	71.53
Level of education of caregiver		
No formal education	37.75 (30.37–45.74)	62.25
Primary	46.10 (38.04–54.37)	53.9
Middle/JHS	33.21 (27.94–38.95)	66.79
SHS/commercial	33.33 (24.09–44.06)	66.67
Tertiary	19.44 (9.55–35.56)	80.56
Primary occupation of caregiver		
Unemployed	38.52 (30.31–47.45)	61.48
Formally employed	21.43 (9.94–40.25)	78.57
Fisherfolks	38.10 (29.32–47.73)	61.9
Trader	34.97 (29.98–40.32)	65.03
Artisan	38.74 (30.14–48.11)	61.26
Community		
Anyanui	29.79 (18.49–44.25)	70.21
Ateteti	34.78 (22.51–49.48)	65.22
Anyako	43.40 (34.29–52.97)	56.6
Opetekwei	23.67 (17.85–30.67)	76.33
Mumford	41.36 (36.11–46.81)	58.64

The prevalence of diarrhoea in the past 2 weeks was 36% (95% CI 33–40%). The proportions of diarrhoea cases across the communities were found to be significantly different. Children in Anyako reported the highest percentage 43.4% (34.29–52.97) and Opetekwei reported the least percentage of diarrhoea cases 23.67 (17.85–30.67).

### WASH and Health practices of Caregivers

More than 60% of the children under five years were reported to be fully vaccinated in all the communities and had received rotavirus vaccine. The health facility was the main site for treatment of household members who were not well. Almost all (98.7%: 684/693) used water from an improved water source for cooking and drinking (Table 3).

**Table 3:** Health practices and WASH practices of caregivers in study sites, 2021

Variable	Anyanui N = 47	Ateteti N = 46	Anyako N = 106	Opetekwei N = 170	Mumford N = 324	Total N = 693
Diarrhoea in past 2 weeks						
No	33 (70.2)	30 (65.2)	60 (56.6)	130 (76.5)	190 (58.6)	443 (63.9)
Yes	14 (29.8)	16 (34.8)	46 (43.4)	40 (23.7)	134 (41.4)	250 (36.1)
Vaccination status of < 5 years						
Not vaccinated	10 (21.3)	6 (13.0)	24 (22.6)	30 (17.6)	115 (35.5)	185 (26.7)
Fully vaccinated	37 (78.7)	40 (87.0)	82 (77.4)	140 (82.4)	209 (64.5)	508 (73.3)
Household treatment site						
Health facility	43 (91.5)	45 (97.8)	104 (98.1)	143 (84.1)	284 (87.7)	619 (89.3)
Other care	4 (8.5)	1 (2.2)	2 (1.9)	27 (15.9)	40 (12.3)	74 (10.7)
Source of health information						
Health facility	39 (83.0)	38 (82.6)	89 (84.0)	109 (64.1)	225 (69.4)	500 (72.2)
Other people	3 (6.4)	3 (6.5)	7 (6.6)	25 (14.7)	40 (12.3)	78 (11.2)
Media	5 (10.6)	5 (10.9)	10 (9.4)	36 (21.2)	59 (18.2)	115 (16.6)
Water source for household**						
Unimproved	1 (2.1)	3 (6.5)	5 (4.7)	0 (0.0)	0 (0.0)	9 (1.3)
Improved	46 (97.9)	43 (93.5)	101 (95.3)	170 (100)	324 (100)	684 (98.7)
Household Toilet facility**						
Unimproved	8 (17.0)	0 (0.0)	10 (9.4)	2 (1.2)	162 (50.0)	182 (26.3)
Improved	39 (83.0)	46 (100)	96 (90.6)	168 (98.8)	162 (50.0)	511 (73.7)
Household water storage**						
Plastic container	36 (76.6)	33 (71.7)	72 (67.9)	163 (95.9)	315 (97.2)	619 (89.3)
Earthern ware container	6 (12.8)	12 (26.1)	7 (6.6)	0 (0.0)	1 (0.3)	26 (3.8)
Tank gallon	5 (10.6)	1 (2.2)	27 (25.5)	7 (4.1)	8 (2.5)	48 (6.9)
Waste disposal site**						
Nearby container	13 (27.7)	2 (4.3)	2 (1.9)	37 (21.8)	128 (39.5)	182 (26.3)
Open spaces	19 (40.4)	33 (71.7)	96 (90.6)	53 (31.2)	175 (54.0)	376 (54.3)
Burn or bury	15 (31.9)	11 (23.9)	8 (7.5)	11 (6.5)	21 (6.5)	66 (9.5)
Collection service	0 (0.0)	0 (0.0)	0 (0.0)	69 (40.6)	0 (0.0)	69 (9.9)

**p < 0.001

### Factors associated with diarrhoea occurrence among children under five years

The community a child lived in was found to be associated with diarrhoea occurrence. Children in Mumford were 1.7 times more likely to have diarrhoea compared to those in Opetekwei (1.71 (1.20–2.46). a child in Anyako was 1.77 times more likely to have diarrhoea compared to a child in Opetekwei (aRR 1.77, 95% CI 1.18–2.64). Other demographic factors were not found to be significant (Tables 4, 5).

**Table 4:** Association between diarrhoea and background characteristics of caregivers in study sites, 2021

	Crude	Adjusted
Variable	PR (95% CI)	PR (95% CI)
Caregiver age		
18–29 years	1.00	1.00
30–39 years	0.80 (0.64–0.99)	0.84 (0.66–1.07)
40–49 years	0.71 (0.51–0.99)	0.70 (0.49–1.01)
50+	0.84 (0.59–1.20)	0.85 (0.59–1.21)
Number in household		
Less than 5	1.00	1.00
5–10	1.12 (0.91–1.38)	1.13 (0.91–1.40)
More than 10	1.28 (0.76–2.15)	1.21 (0.70–2.06)
Wealth index		
Poorest	1.00	1.00
Poor	0.95 (0.70–1.29)	0.99 (0.72–1.34)
Middle	0.96 (0.71–1.29)	1.00 (0.73–1.36)
Rich	1.05 (0.79–1.40)	1.21 (0.90–1.62)
Richest	0.74 (0.53–1.03)	1.24 (0.84–1.84)
Level of education of caregiver		
No formal education	1.00	1.00
Primary	1.22 (0.93–1.60)	1.11 (0.83–1.49)
Middle/JHS	0.88 (0.68–1.15)	0.86 (0.64–1.15)
SHS/commercial	0.88 (0.61–1.27)	0.94 (0.62–1.43)
Tertiary	0.52 (0.26–1.03)	0.62 (0.28–1.40)
Primary occupation of caregiver		
Unemployed	1.00	1.00
Formally employed	0.56 (0.26–1.17)	0.87 (0.37–2.04)
Fisherfolks	0.99 (0.71–1.38)	0.93 (0.65–1.33)
Trader	0.91 (0.69–1.19)	0.91 (0.68–1.20)
Artisan	1.01 (0.73–1.39)	1.06 (0.76–1.47)
Community		
Anyanui	1.26 (0.75–2.11)	1.23 (0.73–2.09)
Ateteti	1.47 (0.91–2.37)	1.52 (0.89–2.61)
Anyako	1.83 (1.30–2.60)**	1.77 (1.18–2.64)**
Opetekwei	1.00	1.00
Mumford	1.74 (1.29–2.36)**	1.71 (1.20–2.46)**

**p < 0.001, PR: Prevalence ratio

**Table 5:** Association between diarrhoea and WASH and health practices of caregivers in study sites, 2021

Factor	Crude PR (95% CI)	Adjusted PR (95% CI)
N		
Vaccination status of < 5 years		
Not vaccinated	1.00	1.00
Fully vaccinated	0.67(0.54–0.80)*	0.68 (0.55–0.84)*
Household treatment site		
Health facility	1.00	1.00
Other care	0.93(0.66–1.30)	0.92 (0.64–1.33)
Source of health information		
Health facility	1.00	1.00
Other people	1.07(0.79–1.45)	1.18 (0.85–1.64)
Media	0.96(0.73–1.27)	1.11(0.834–1.47)
Water source for household		
Unimproved	1.00	1.00
Improved	0.54(0.33–0.86)*	0.59(0.34–1.03)
Household Toilet facility		
Unimproved	1.00	1.00
Improved	0.88(0.711–1.09)	1.12(0.88–1.44)
Household water storage		
Plastic container	1.00	1.00
Earthern ware container	1.05(0.64–1.72)	1.09(0.66–1.78)
Tank gallon	0.75(0.47–1.21)	0.71(0.43–1.17)
Waste disposal site		
Nearby container	1.00	1.00
Open spaces	1.17(0.92–1.48)	1.18(0.91–1.52)
Burn or bury	1.03(0.70–1.52)	1.27(0.85–1.90)
Collection service	0.77(0.50–1.21)	1.3(0.74–2.33)

*p < 0.05, PR: Prevalence ratio

Children who were fully vaccinated had 0.32 reduced likelihood of having diarrhoea compared to those who were not aRR 0.68 (95% CI 0.55–0.84). All other variables were not found to be significantly associated to diarrhoea from the binary and multiple regression models.

## Discussion

This study assessed factors associated to diarrhoea in selected coastal communities in the eastern coast of Ghana. The study found that the diarrhoea prevalence was high. Almost four out of ten children under five years in the communities had experienced diarrhoea in the past two weeks. Other studies have reported lower prevalence of diarrhoea among children under 5 years even in settings with unimproved WASH conditions [24–27]. The high prevalence could be attributed to the seasonal nature of the disease. Diarrhoea generally is known for its seasonal nature, and peaks in extreme weather conditions [28–30]. Interpretation of diarrhoea prevalence therefore needs to be done in context of time to provide a more accurate picture of the situation. Even in considering seasonal variations, this high prevalence is still be worth noting.

The only factor found to be associated with the occurrence of diarrhoea in this study was vaccination of the children under five years. Fully vaccinated children were less likely to get diarrhoea compare to those who have never been vaccinated or were not fully vaccinated. Rotaviruses have been found to be one of the leading pathogens causing diarrhoea among children under five. Each year, rotavirus was reported to attributed to about 2 million cases [31]. After the introduction of the rotavirus vaccine into the childhood immunization scheme, the prevalence of diarrhoea had generally declined in most places[32]. A study in Fuji recorded an 87% decline in hospitalizations after introduction of the rotavirus vaccine in the country[33]. This finding, therefore confirms the need for children to be appropriately vaccinated to help build their immunity against diarrhoea and other diseases of public health concern.

Another finding from the study was the health facility serving as the communities’ major point of call when seeking treatment or health education. This finding shows the confidence the communities have in their local health facilities. For any health system to be able to make an impact in a population, there is the need for the trust of the people to be obtained (9,10) these studies have shown that in settings where the health system works with the community, they are able to achieve many health targets.

Majority of the caregivers reported using improved Water and sanitation sources. Nine in ten of the respondents reported that their drinking and cooking water was from an improved source. For coastal communities, the water, hygiene and sanitation structures and use reported were relatively high. Also, their use reveals the communities’ acceptance of the WASH interventions available to them. Implementation of improved WASH in communities has been one of the main interventions over the years for the reduction of diarrhoeal related morbidities [15, 24, 37–39]. These interventions have been effective in a number of settings and led to the improvement of the health of the community as a whole, including children under five years.

The study finding on high diarrhoea morbidity in a setting with improved WASH and good health seeking behaviour however is contrary to several studies which have stated that the presence of improved WASH interventions and health seeking behaviour translate into the reduction in diarrhoea prevalence [14, 40–43]. This finding reveals that it might not be enough to conclude that the presence of WASH and other health promotion interventions alone can contribute to reduction in the prevalence of diarrhoea in a community.

Reporting a clinical trial in Kenya, Null revealed that improved WASH interventions alone were not enough to reduce diarrhoea among children under five years [44]. A recent assessment of three major large scale clinical trials on the effect of WASH on childhood diarrhoea in different settings reveals that relying solely on WASH interventions as way of reducing diarrhoea might be a challenge since it doesn’t always happen that way[45].

Watson and colleagues in their paper on evaluations on WASH interventions suggest the need to reconsider diarrhoea morbidity as a primary outcome for these interventions due to its peculiarities which could lead to misclassification and false estimation of the effects of interventions [46]. In the wake of questioning the effectiveness of WASH interventions using the prevalence of diarrhoea, there is the need to relook at the traditional assessment approaches used. Eisenberg proposes the addition of other assessments which take in consideration the transmission pathway of diarrhoea especially in settings with improved WASH interventions [47]. This is likely to help in identifying other underlying factors which might have been over looked.

Since diarrhoea continues to persists in low-resourced settings with improved WASH such as in this current study, some schools of thoughts also suggest the need to consider assessing other determinants aside WASH as a way to manage the diarrhoea menace. To buttress this line of thought, a study in Ethiopia assessed diarrhoea determinants in households with improved WASH structures and found measles vaccination, living in a particular geographical location and a child being 13 -24 months as factors associated with developing diarrhoea[48].

A major limitation to this study is the use of a cross-sectional study design in the assessment. Given that cross-sectional studies are unable to capture seasonality, the diarrhoea might vary across seasons. Nevertheless, findings form the study bring out the need to conduct such assessments periodically to provide relevant information on the diarrhoea situation in vulnerable settings such as coastal areas.

## Conclusion

In spite of high diarrhoea prevalence, good WASH practices and health seeking practices reported were generally high. Also, fully vaccinated children had a relatively lower prevalence of diarrhoea. This findings confirms that reported WASH and health seeking practices alone may not be able to adequately predict the underlying causes of diarrhoea especially in a coastal setting. There is the need to consider indepth assessments which consider the multifacted diarrhoeal factors and even the efficacy of the existing interventions. Further investigation may be needed to understand the issue, identify gaps and ways to address them so that interventions targets can be met. We recommend in-depth analysis of the use of water and sanitation facilities in these settings and further research to understand the reasons for the observed diarrhoea prevalence observed.

## Declarations

### Availability of data and materials

All data used for the statistical analysis are available from the corresponding author upon reasonable request.
